# Macrophage-Specific Connexin 43 Knockout Protects Mice from Obesity-Induced Inflammation and Metabolic Dysfunction

**DOI:** 10.3389/fcell.2022.925971

**Published:** 2022-06-21

**Authors:** Cheoljun Choi, Abhirup Saha, Seungchan An, Yoon Keun Cho, Heeseong Kim, Minsoo Noh, Yun-Hee Lee

**Affiliations:** College of Pharmacy and Research Institute of Pharmaceutical Sciences Seoul National University, Seoul, South Korea

**Keywords:** connexin 43, macrophage, adipose tissue remodeling, obesity, inflammation

## Abstract

Adipose tissue macrophages are a major immune cell type contributing to homeostatic maintenance and pathological adipose tissue remodeling. However, the mechanisms underlying macrophage recruitment and polarization in adipose tissue during obesity remain poorly understood. Previous studies have suggested that the gap junctional protein, connexin 43 (Cx43), plays a critical role in macrophage activation and phagocytosis. Herein, we investigated the macrophage-specific roles of Cx43 in high fat diet (HFD)-induced pathological remodeling of adipose tissue. Expression levels of Cx43 were upregulated in macrophages co-cultured with dying adipocytes *in vitro*, as well as in macrophages associated with dying adipocytes in the adipose tissue of HFD-fed mice. Cx43 knockdown reduced lipopolysaccharide (LPS)-induced ATP release from macrophages and decreased inflammatory responses of macrophages co-cultured with dying adipocytes. Based on global gene expression profiling, macrophage-specific Cx43-knockout (Cx43-MKO) mice were resistant to HFD-induced inflammatory responses in adipose tissue, potentially *via* P2X7-mediated signaling pathways. Cx43-MKO mice exhibited reduced HFD-induced macrophage recruitment in adipose tissue. Moreover, Cx43-MKO mice showed reduced inflammasome activation in adipose tissues and improved glucose tolerance. Collectively, these findings demonstrate that Cx43 expression in macrophages facilitates inflammasome activation, which, in turn, contributes to HFD-induced metabolic dysfunction.

## 1 Introduction

Obesity is characterized by abnormal fat accumulation, and its growing prevalence is closely associated with a high incidence of chronic metabolic diseases, including type 2 diabetes and cardiovascular diseases (Srivastava and Apovian, 2017). Adipose tissue dysfunction is one of the major contributing factors to the pathogenesis of obesity-related metabolic diseases (Srivastava and Apovian, 2017). Typically, the abnormal expansion of adipose tissue appears to be accompanied by the recruitment of pro-inflammatory immune cells, and these chronic inflammatory responses can lead to insulin resistance in multiple metabolic organs (Reilly and Saltiel, 2017).

Macrophages are a major immune cell type present in adipose tissues (Epelman et al., 2014). Notably, changes in macrophage phenotype and polarization status can contribute to the development of obesity-related diseases (Lee et al., 2010). For example, adipose tissue of individuals with obesity recruits pro-inflammatory macrophages (Reilly and Saltiel, 2017), whereas anti-inflammatory macrophages participate in tissue remodeling and homeostasis of adipose tissue (White and Ravussin, 2019). However, precise mechanisms underlying macrophage recruitment to adipose tissue during obesity need to be comprehensively elucidated.

Connexin, a gap junctional protein, plays a key role in gap junctional communication between cells, allowing the conduction of small molecules. For example, gap junction-mediated coupling of electrical stimuli between cells, such as cardiomyocytes, is critical for propagating electrical action potential (Evans and Martin, 2002). In addition to gap junctional channels, connexins can form hemichannels. Previous studies have indicated that connexin 43 (Cx43) hemichannels can induce ATP release from inflammatory cells and consequently regulate the autocrine activation of macrophages via ATP signaling (Dosch et al., 2019).

In the present study, we investigated the pathophysiological role of Cx43 using a co-culture system of dying adipocytes with adipose tissue macrophages and a macrophage-specific Cx43 knockout (KO; Cx43-MKO) mouse model. Interestingly, Cx43 expression was upregulated in macrophages co-cultured with dying adipocytes, as well as in adipose tissue of mice fed a high-fat diet (HFD). Cx43 knockdown reduced extracellular ATP levels and inflammatory responses in macrophages *in vitro*. *In vivo*, Cx43-MKO mice were protected against HFD-induced inflammation and glucose intolerance.

## 2 Materials and Methods

### 2.1 Animals

All animal protocols were reviewed and approved by the Institutional Animal Care and Use Committee at Seoul National University (SNU-181120–3, SNU-191204-4–1). All animal experiments were conducted in strict compliance with the guidelines for humane care and use of laboratory animals specified by the Ministry of Food and Drug Safety. Male mice were used for experiments. Mice were housed at 22 ± 1°C and maintained on a 12-h light/12-h dark cycle with free access to food and water at all time. C57BL/6 mice were purchased from Central Lab. Animal Inc. *Csf1r*-CreER (Qian et al., 2011) (stock# 019,098, FVB-Tg (Csf1r-cre/Esr1*)1Jwp/J), and *Gja1* ^flox/flox^ (stock #008039, B6.129s7-Gja1tm1Dlg/J) (Rios et al., 2001) mice were purchased from the Jackson Laboratory. *Csf1r-*CreER mice and *Gja1* ^flox/flox^ mice were crossed to produce inducible macrophage-specific connexin 43 (Cx43) KO mice (Csf1r-CreER/Gja1 ^flox/flox^: Cx43-MKO mice). For wild type (WT) control, Gja1 floxed mice (Gja1^flox/flox^) without CreER were used. For Cre recombination, Cx43-MKO mice and WT controls were treated with tamoxifen (75 mg/kg/day, Cayman) dissolved in sunflower oil by oral gavage for five consecutive days. Experiments were started 10 days after the last dose of tamoxifen. For HFD experiment, Cx43-MKO and WT mice were fed a 60% fat diet (Research Diet) or normal chow diet (NCD) for 8 weeks (n = 6 per condition: WT-NCD, WT-HFD, Cx43-MKO-NCD, Cx43-MKO-HFD). For intraperitoneal glucose tolerance test and insulin tolerance test, mice were given D-glucose (2 g/kg body weight, 200 mg/ml, Sigma) and insulin (0.75 U/kg body weight, Sigma) by intraperitoneal injection, respectively, and blood glucose levels were measured at indicated time points (*n* = 6 per condition)

### 2.2 Western Blot Analysis

Western blot analysis was performed as described previously (Lee et al., 2015). Briefly, protein was extracted in RIPA buffer (Thermo Fisher) containing protease inhibitors (Sigma) and phosphatase inhibitors (Roche). Resolved proteins were transferred to polyvinylidene difluoride (PVDF) membranes, the membranes were incubated with blocking buffer (5% skim milk Tris Buffered Saline with Tween® 20), primary and secondary antibodies. The antibodies used for western blot are listed in Supplementary Table S1.

### 2.3 Gene Expression Analysis

Quantitative PCR was performed as described previously (Lee et al., 2015). Briefly, RNA was extracted using TRIzol® reagent (Invitrogen), and was reverse transcribed using a cDNA synthesis kit (Applied Biosystems). 100ng of cDNA was subjected to quantitative polymerase chain reaction (qPCR) in 20-μL reaction volumes (iQ SYBR Green Supermix, Bio-Rad) with 100 nM primers. qRT-PCR was performed using SYBR Green dye and CFX Connect Real-time system (Bio-Rad) for 45 cycles and fold change for all samples was calculated by using the 2−ΔΔCt method. The primers used for qPCR are listed in Supplementary Table S2. Peptidylprolyl Isomerase A (PPIA) was used as a housekeeping gene for mRNA expression analysis. Primers used for qRT-PCR were described previously (Kwon et al., 2016; Lee et al., 2016).

### 2.4 RNA Sequencing Analysis

RNA sequencing (RNA-seq) analyses were performed as previously described. Briefly, Trizol reagent (Invitrogen) was used for total RNA extraction of gWAT, according to the manufacturer’s instruction. RNA integrity number (RIN), rRNA ratio, and concentration of samples were verified on an Agilent Technologies 2100 Bioanalyzer (Agilent Technology) using a DNA 1000 chip. For RNA-seq analysis, cDNA libraries were constructed with the TruSeq mRNA Library Kit using 1 mg of total RNA. The total RNA was sequenced by the NovaSeq 6000 System (Macrogen).

Raw sequenced reads were trimmed for adaptor sequence, and then HISAT2 v2.1.0 was used to map the trimmed reads to the reference genome. After read mapping transcript assembly was performed with Stringtie v1.3.4 days and calculated raw transcription profiles as a fragment per Kilobase of transcript per Million mapped reads (FPKM) for each gene and each sample (Fiechter et al., 2021).

The DESeq2 package (v.1.24.0) was used to normalize read counts and determine differentially expressed genes (DEGs) among samples (Love et al., 2014). DEGs were defined by a cut-off values of 1.5-fold change and *p* value <0.05. Principal component analysis (PCA) was performed for selection of variable genes and dimensionality reduction using *prcomp* function in R v.3.6.1 (R Core Team, Vienna, Austria). The Hallmark gene sets (h.all.v7.5.symbols) were used for the Gene Set Enrichment Analysis (GSEA, v.4.0.3) by using the list of genes pre-ranked by principal component (PC) one loadings (Subramanian et al., 2005). Module scores were obtained by calculating the average of Z-normalized expression levels of genes in each gene set. Using the lists of DEGs, gene ontology (Subramanian et al.) enrichment analysis was performed with gene ontology biological process enrichment analysis as previously described (Ahn et al., 2019) and gprofiler2 v.0.2.0 package (Raudvere et al., 2019).

### 2.5 Histology

Adipose tissue was processed for histological sections, and 5 μm-thick paraffin sections were subjected to hematoxylin/eosin (H/E) staining or immunohistochemical analysis, as previously described (Lee et al., 2017). Anti-F4/80 antibody (rat, 1; 100, Serotech) and anti-Cx43 antibody (rabbit, 1:100, Cell Signaling) were used for immunohistological analysis. Fluorescence intensity of images was analyzed by using ImageJ software.

### 2.6 SVC and Adipocyte Fractionation and Flow Cytometry

Stromovascular cells (SVC) and adipocytes from gonadal white adipose tissue (gWAT) were fractionated, as previously described (Lee et al., 2012; Lee et al., 2017; Cho et al., 2019). Live cells were processed for cell surface marker staining. Antibodies used for flow cytometry analysis were the following: anti-F4/80-APC and CD11c-BV421 (Biolegends). Anti-F4/80-FITC (Biolegends), anti-Cx43 (rabbit, 1:100, Cell Signaling), and goat anti-Rabbit IgG (H+L) secondary antibody Alexa Fluor^TM^ 594 (rabbit, 1:100, Invitrogen). Analytic cytometry was performed using BD FACSLyric^TM^ (BD Biosciences) flow cytometers. Raw data were processed using FlowJo software (Tree Star). For the identification of cell types in flow cytometry data, at least 10,000 cells were analyzed per sample.

For macrophage isolation, dissociated adipose tissue was fractionated by magnetic cell sorting (MACS) with anti-F4/80-FITC/anti-FITC-microbeads (Miltenyi Biotech).

### 2.7 Cell Cultures

The C3H10T1/2 cells and RAW 264.7 cells (ATCC) were cultured, as previously described (Nykjaer et al., 2004; Kim et al., 2017). For knockdown, siRNA targeting *Gja1* (EMU006781, Sigma) was transfected into RAW 264.7 cells, using INTERFERin (Polyplus). siRNA Universal negative controls were used. Dying adipocytes were obtained by maintaining fully differentiated C3H10T1/2 adipocytes in Dulbecco’s Modified Eagle Medium (DMEM) containing 1 μg/ml insulin for 10 days post-differentiation. For MACS-isolated F4/80+ macrophages from gWAT were cultured at an initial concentration of 1 × 10^5^ cells/ml in growth medium. The co-culture experiments were performed as described previously (Kwon et al., 2016). Briefly, fully differentiated C3H10T1/2 adipocytes (10 days post-differentiation) were trypsinized and counted, and then 1 × 10^5^ cells/ml were added to each well of 12-well dishes that contained macrophage cultures. To test the effects of ATP degradation, apyrase (ATP-diphosphohydrolase, 2U/ml, Sigma) (Kruse et al., 2014) was treated before co-culture, and maintained in the media during co-culture with dying adipocytes. For long-term imaging, differentiated C3H10T1/2 adipocytes were labeled with 4,4-difluoro-5-(2-thienyl)-4-bora-3a, 4a-diaza-s-indacene3-dodecanoic acid (BODIPY 558/568 C12) (Invitrogen), and macrophages or RAW 264.7 cells were labeled with Vybrant DiO Cell-Labeling Solution (Invitrogen) overnight. BODIPY-labeled C3H10T1/2 cells were detached, added to the DiO-labeled macrophages, and then co-cultured for 12 h. To monitor the phagocytosis of the dying adipocytes by the macrophages, live cell imaging was performed every 2 h with Operetta CLS High-Content Analysis System (Perkin Elmer), and the fluorescence intensity of the images was analyzed using Harmony (Perkin Elmer) high-content imaging and analysis software. ATP levels were measured with an ATP Determination Kit (Invitrogen) after stimulating with lipopolysaccharide (LPS) for 30 min (Cauwels et al., 2014), following manufacturer’s instructions.

For immunocytochemistry analysis, the cells were seeded into glass-bottom culture slide and proceeded to immunofluorescence staining, as previously described (Cho et al., 2019). Primary antibody used for staining was anti-Cx43 antibody (rabbit, 1:100, Cell Signaling).

### 2.8 Statistical Analysis

Statistical analyses were performed using GraphPad Prism 7 software (GraphPad Software). Data are presented as mean ± standard errors of the means (SEMs). Statistical significance between two groups was determined by unpaired t-test. Comparisons among multiple groups were performed using a two-way analysis of variance (ANOVA), with Bonferroni post hoc tests to determine *p* values.

### 2.9 Data and Resource Availability

All data generated or analyzed during this study are included in this article or are available from the corresponding authors on request. The raw RNA-seq data have been deposited in Gene Expression Omnibus (GEO) (GSE 204794).

## 3 Results

### 3.1 Macrophage Expression of Cx43 Was Upregulated in Gonadal White Adipose Tissue of HFD-Fed Mice

**FIGURE 1:**
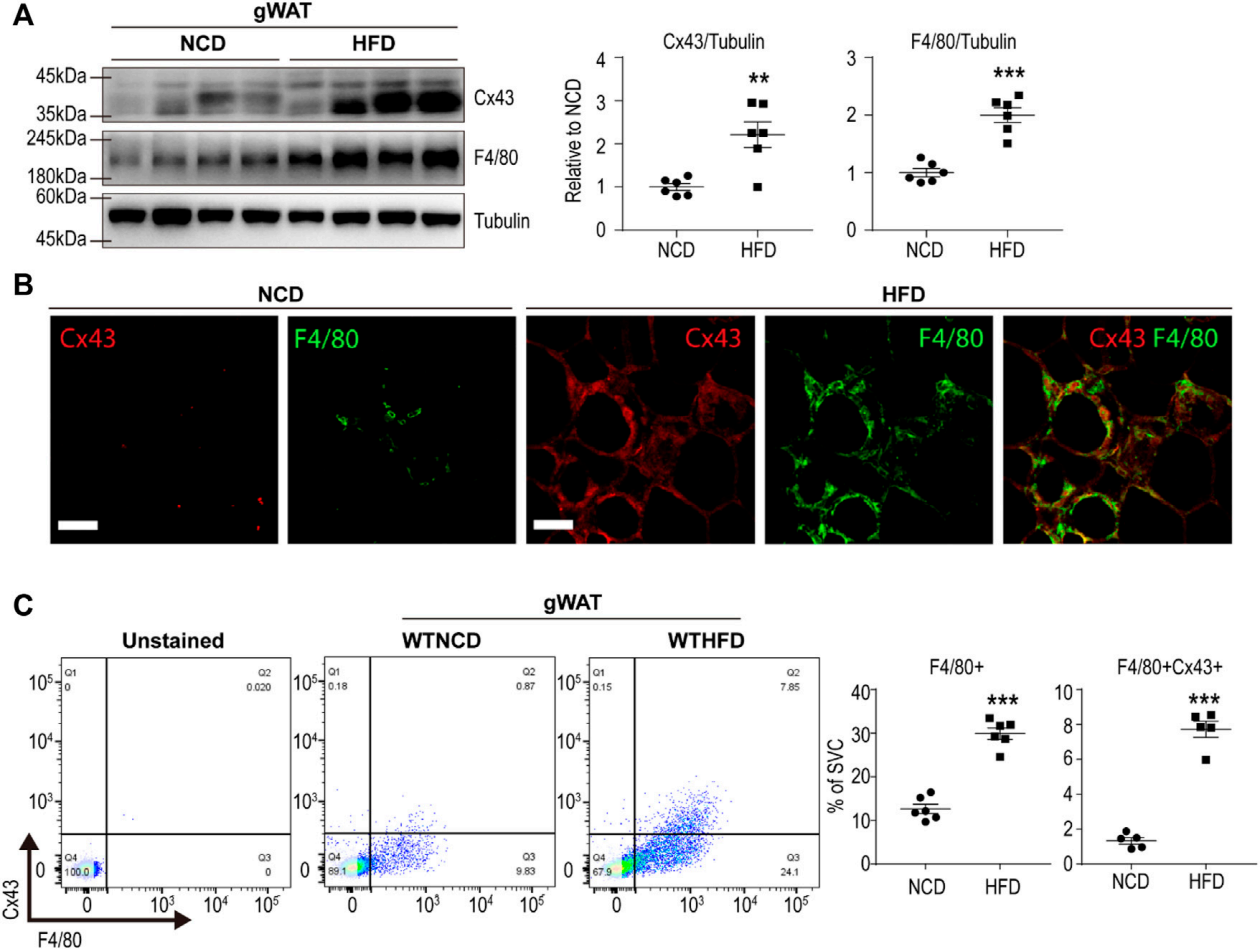
Cx43 is upregulated in gonadal white adipose tissue macrophages of mice by high-fat diet feeding **(A)** Immunoblot analysis of Cx43 and F4/80 expression in gonadal white adipose tissue (gWAT) of mice fed normal chow diet (NCD) or high-fat diet (HFD) for 8 weeks (*n* = 6) **(B)** Immuno-fluorescence images of paraffin sections of gWAT of mice fed NCD or HFD, stained for Cx43 and F4/80 **(C)** Flow cytometric analysis of stromovascular cells (SVCs) obtained from gWAT of WT mice fed with NCD or HFD for 8 weeks (n = 6). Unpaired, two-tailed t-tests (**p < 0.01, ***p < 0.001), each point represents biological replicate. Data are presented as mean ± S.E.M.

We examined Cx43 expression levels in gonadal white adipose tissue (gWAT) of mice fed a HFD for 8 weeks. Western blot analysis revealed that HFD-induced Cx43 upregulation in gWAT (Figure 1A). In addition, the immunohistochemical analysis further confirmed that macrophages surrounding adipocytes (i.e., crown-like structures) expressed high levels of Cx43 (Figure 1B). Flow cytometry analysis of SVCs of gWAT also indicated that HFD feeding induced F4/80+ macrophage recruitment and increased Cx43 expression in F4/80+ cells (Figure 1C).

### 3.2 Macrophages Co-cultured With Dying Adipocytes Upregulated Cx43 Expression

**FIGURE 2:**
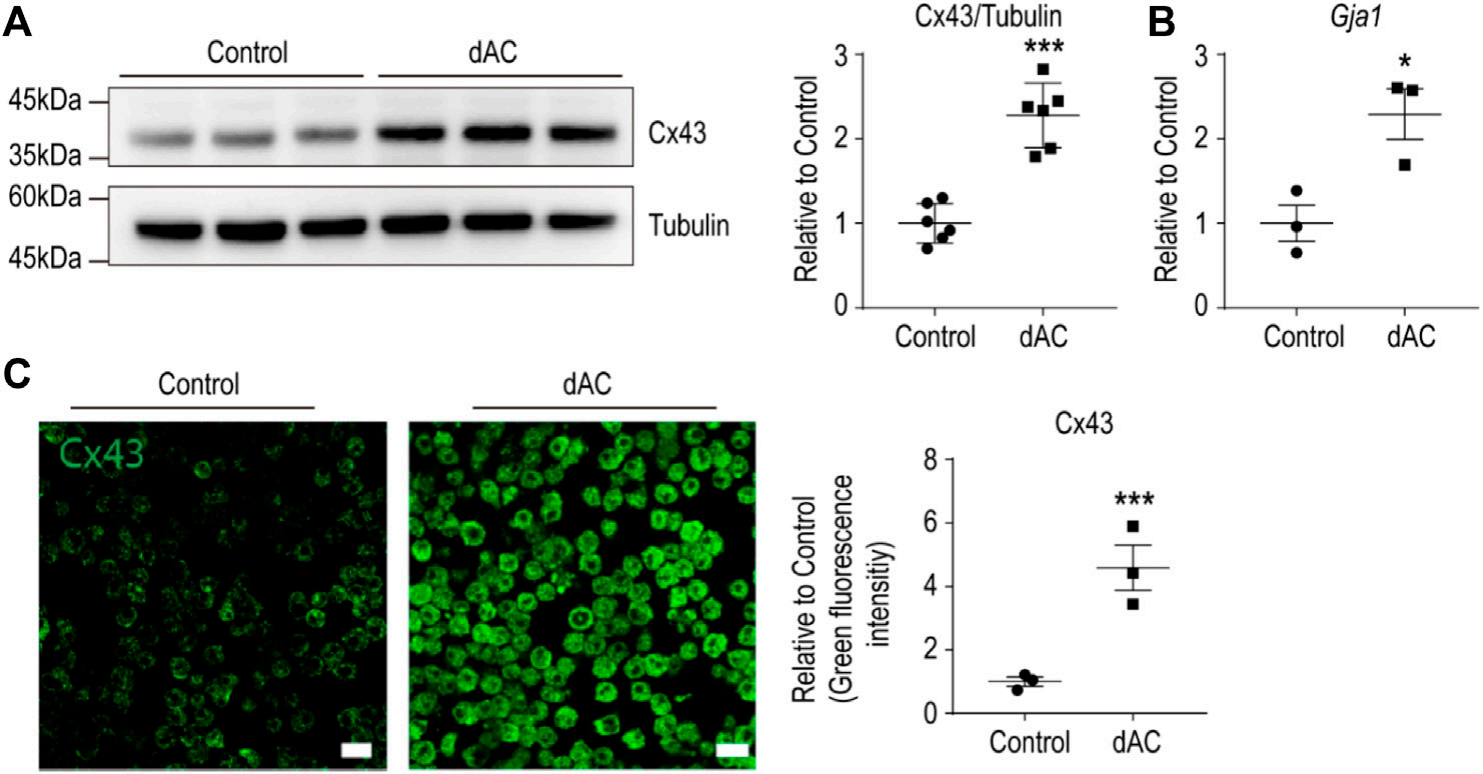
Macrophages co-cultured with dying adipocytes (dAC) upregulate Cx43 expression **(A–B)** Immunoblot analysis of Cx43 protein **(A)** and qPCR analysis of *Gja1* transcript levels **(B)** in F4/80^+^ macrophages isolated from gWAT co-cultured with dying adipocytes (dAC) for 24 h (A: *n* = 6, B: *n* = 3) **(C)** Immunofluorescence images of Cx43 staining in F4/80^+^ macrophages from gWAT co-cultured with or without dying adipocytes (dAC) for 24 h (*n* = 3). Unpaired, two-tailed t-tests (*p < 0.05, ***p < 0.001), each point represents biological replicate. Data are presented as mean ± S.E.M.

HFD-induced adipose tissue remodeling is characterized by adipocyte hypertrophy, adipocyte death, and macrophages surrounding damaged/dying adipocytes (Strissel et al., 2007). To identify the molecular features of adipose tissue macrophages involved in HFD-induced adipose tissue remodeling, we established *in vitro* system by co-culturing macrophages with dying adipocytes (dAC) (Cho et al., 2019). As previously described (Lee et al., 2016), dying adipocytes were obtained from prolonged cultures of adipocytes differentiated from C3H10T1/2 cells. MACS-isolated adipose tissue macrophages were cultured with the dying adipocytes (Anand et al., 2008). We confirmed the upregulation of Cx43 expression in macrophages using immunoblotting, quantitative PCR, and immunostaining analysis (Figures 2A–C).

### 3.3 Cx43 Knockdown Reduced Inflammatory Response of Macrophages Co-cultured With Dying Adipocytes

**FIGURE 3:**
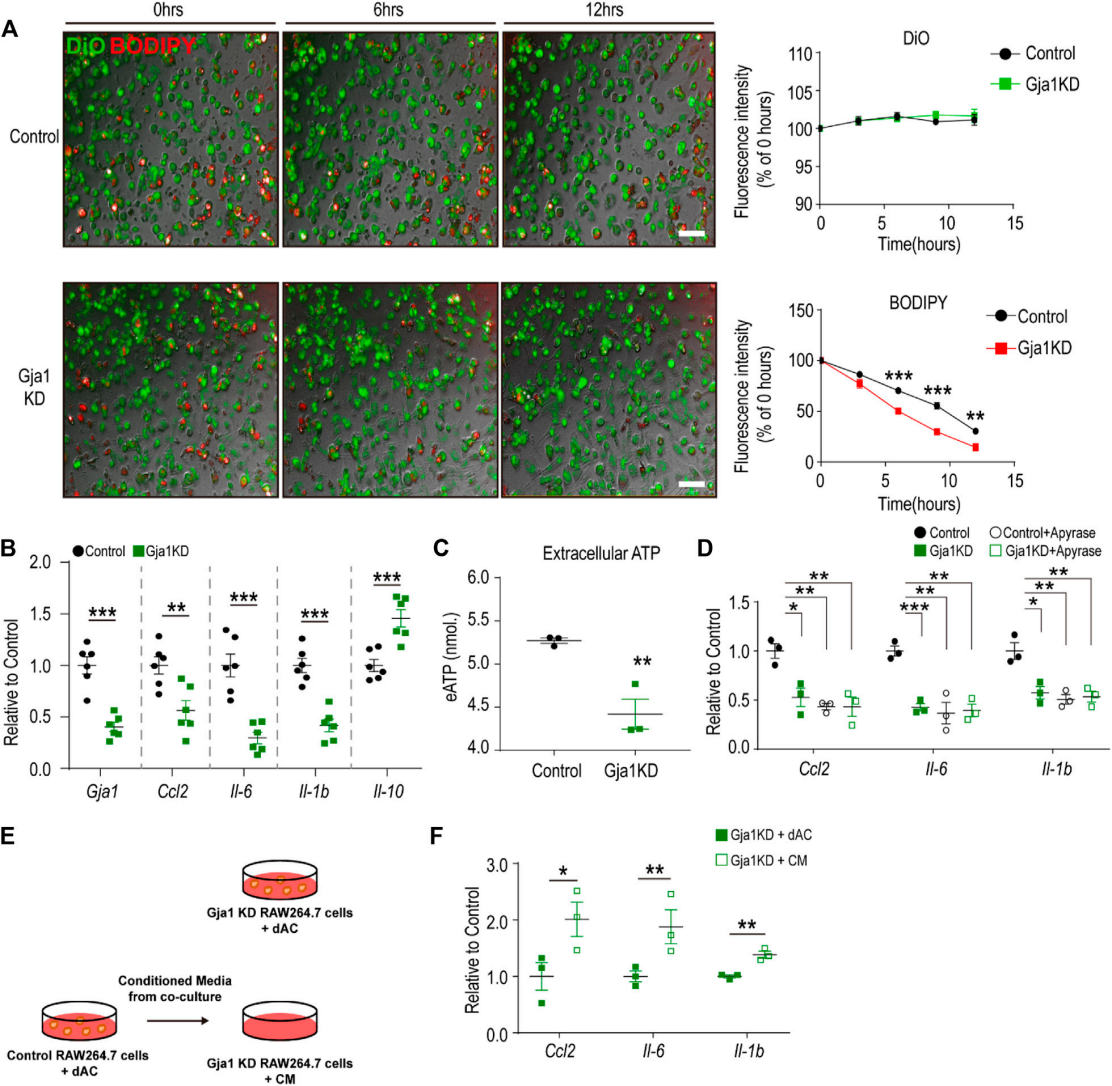
Cx43 knockdown reduces inflammatory responses of macrophages co-cultured with dying adipocytes **(A)** Live imaging of RAW264.7 cells transfected with *Gja1* siRNA or controls and co-cultured with dying adipocytes (dACs) for 12 h. Adipocytes were tagged with C12-BODIPY (red) and macrophages were stained with DiO (Green) (*n* = 3) **(B)** qPCR analysis of *Gja1* and inflammatory cytokine markers in RAW264.7 cells transfected with *Gja1* siRNA or controls and co-cultured with dACs for 24 h (*n* = 6) **(C)** Extracellular ATP level in lipopolysaccharide (LPS)-stimulated RAW 264.7 macrophages treated with *Gja1* siRNA or control for 30 min (*n* = 3) **(D)** qPCR analysis of inflammatory cytokines in RAW264.7 cells transfected with *Gja1* siRNA or controls, and co-cultured with dACs in the presence or absence of apyrase (2U/ml) (*n* = 3) **(E)** Schematic diagram illustrating the experimental method used for F. Conditioned media obtained from control RAW264.7 cells co-cultured with dACs were transferred into *Gja1*KD RAW264.7 cells **(F)** qPCR analysis of inflammatory cytokine markers in Gja1 KD RAW264.7 cells co-cultured with dACs (GjaKD + dAC) or exposed to the conditioned media obtained from the control co-cultures (Gja1KD + CM) (*n* = 3). Unpaired, two-tailed t-tests (*p < 0.1, **p < 0.01, ***p < 0.001). Each point represents biological replicate. Data are presented as mean ± S.E.M.

Next, we investigated the effects of Cx43 knockdown in macrophages co-cultured with dying adipocytes. We performed *Gja1* (encoding Cx43) siRNA knockdown (KD) in RAW264.7 cells. For visualization, we labeled adipocytes with BODIPY and macrophages with DIO dye (Lee et al., 2016). Using this co-culture study, we revealed that Gja1KD increased the clearance of dying adipocytes in macrophages (Figure 3A). Interestingly, Gja1KD decreased the expression of pro-inflammatory markers and increased the expression of genes involved in anti-inflammatory responses (M2 macrophage markers) (Figure 3B).

Previous studies have suggested that Cx43 plays a critical role in the pro-inflammatory activation of macrophages by facilitating the release of ATP and regulating extracellular ATP signaling. Thus, we examined ATP levels in LPS-treated Gja1KD macrophages and found that Gja1KD reduced extracellular ATP levels (Figure 3C). Treatment with apyrase decreased the expression levels of pro-inflammatory cytokines by degrading extracellular ATP (Figure 3D). In addition, we tested the effects of conditioned media obtained from control RAW264.7 cells with dAC (Figure 3E). Data indicated that the conditioned media were sufficient to increase pro-inflammatory cytokines in Gja1KD RAW264.7 cells (Figure 3F), indicating that ATP released through Cx43 hemichannels in the media was a critical signal to induce inflammatory responses.

### 3.4 Global Transcriptomic Analysis Indicated That Macrophage-specific Cx43 KO Reduced Macrophage Recruitment and Inflammation in Adipose Tissue of HFD-Fed Mice

**FIGURE 4:**
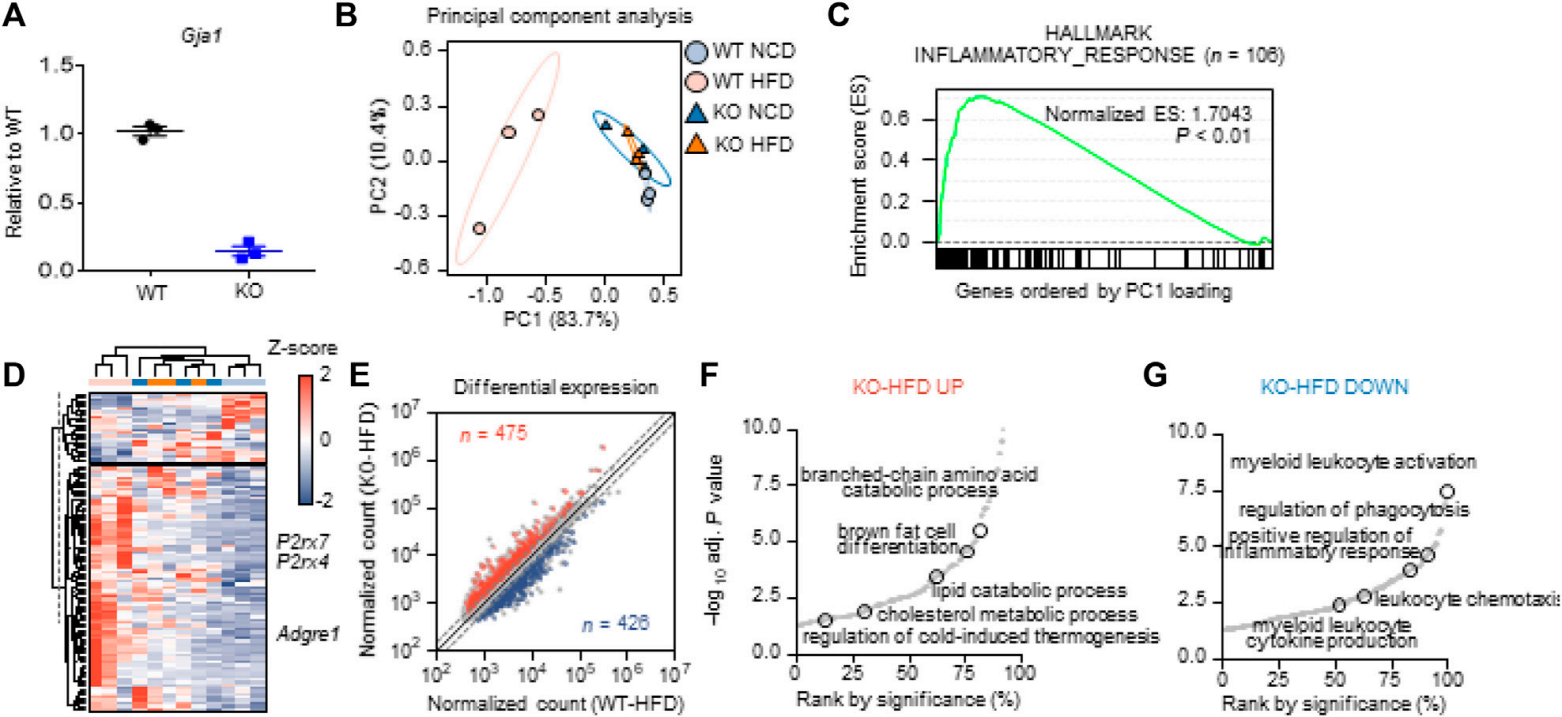
Macrophage-specific Cx43 KO reduced macrophage recruitment andpro-inflammatory responses in adipose tissue of mice fed HFD **(A)** qPCR analysis of *Gja1* in MACS-isolated F4/80+ macrophages from gonadal white adipose tissue (gWAT) of WT and Cx43 MKO mice (*n* = 3) **(B)** Principal component analysis based on the expression levels of 9,895 variable genes for four groups (WT-NCD, KO-NCD, WT-HFD, KO-HFD). PC1 and PC2 values were used for 2-D representation **(C)** GSEA showing enrichment in inflammatory response (*n* = 106) by genes largely contributed to PC1 (*p* < 0.01). Among the variable genes ordered according to PC1 loading, genes associated with the inflammatory response were marked with a black vertical line **(D)** Heatmap showing expression levels of genes associated with inflammatory response **(E)** Differential expression analysis between WT-HFD and KO-HFD. The normalized counts of each group were plotted, and DEGs satisfying fold change >1.5 and *p* < 0.05 were presented in red and blue in case of up-regulated and down-regulated, respectively. The number of DEGs were labeled **(F)** Enrichment analysis using DEGs up-regulated in KO-HFD compared with WT-HFD **(G)** Enrichment analysis using DEGs down-regulated in KO-HFD compared with WT-HFD. Each dot represents significantly enriched gene set and selected pathways were labeled.

To investigate the role of Cx43 expression *in vivo*, we used Cx43-MKO mice. Cx43 floxed mice were crossed with *Csf1r*_CreER mice to delete Cx43 expression in a macrophage-specific manner. After tamoxifen induction, macrophage-specific KO was confirmed by qPCR analysis of MACS-isolated macrophages from the adipose tissue (Figure 4A).

We next aimed to characterize the effects of Cx43-MKO on the molecular phenotype of adipose tissue in an unbiased manner. Accordingly, we performed RNA-seq analysis of gWAT from wild-type (WT) and Cx43-MKO mice fed a HFD or normal chow diet (NCD). Principal component analysis (PCA) based on the expression levels of 9,895 variable genes indicated that the WT-HFD group was characterized by a transcriptional pattern distinct from the other groups (WT-NCD, KO-NCD, and KO-HFD) (Figure 4B). On listing genes ordered according to their contribution levels to PC1 was subjected to Gene Set Enrichment Analysis (GSEA), eight out of 24 hallmark gene sets were significantly affected (Supplementary Table S3). In HFD-fed WT mice, the hallmark inflammatory response was one of the most significantly enriched pathways (*p* < 0.01) in gWAT (Figure 4C). The average expression of genes associated with the inflammatory response was positively regulated in the WT-HFD group; this was not observed in the other groups (Supplementary Figure S1). In addition, PC1 showed a high correlation with the average expression of genes associated with the inflammatory response, thereby confirming that it is a critical biological process differentiating the WT-HFD group from other groups (Supplementary Figure S1). The heatmap indicated that among these 106 genes, 83 genes, including *Adgre1*, *P2rx7*, and *P2rx4*, were highly expressed in the WT-HFD group, while 23 genes were downregulated (Figure 4D). In the DEG analysis, HFD upregulated 821 genes and downregulated 985 genes in WT mice but significantly affected only 130 genes in KO mice, thus indicating that Cx43-MKO counteracted the effect of HFD (Supplementary Figures S2A, S2D).

In addition, we analyzed differentially expressed genes between the WT-HFD and KO-HFD groups to determine the effect of Cx43 KO. We found that 475 genes were upregulated, whereas 426 genes were downregulated (Figure 4E). In the KO-HFD group, pathways associated with lipid metabolic (catabolic) processes were enriched (Figure 4F, Supplementary Figure S2C). Pathways associated with leukocyte activation and chemotaxis were enriched in the WT-HFD group (Figure 4G, Supplementary Figure S2C).

### 3.5 Cx43 KO Protects Mice From HFD-Induced Metabolic Dysfunction, Partly by Reducing the Inflammasome Signaling Pathway

**FIGURE 5:**
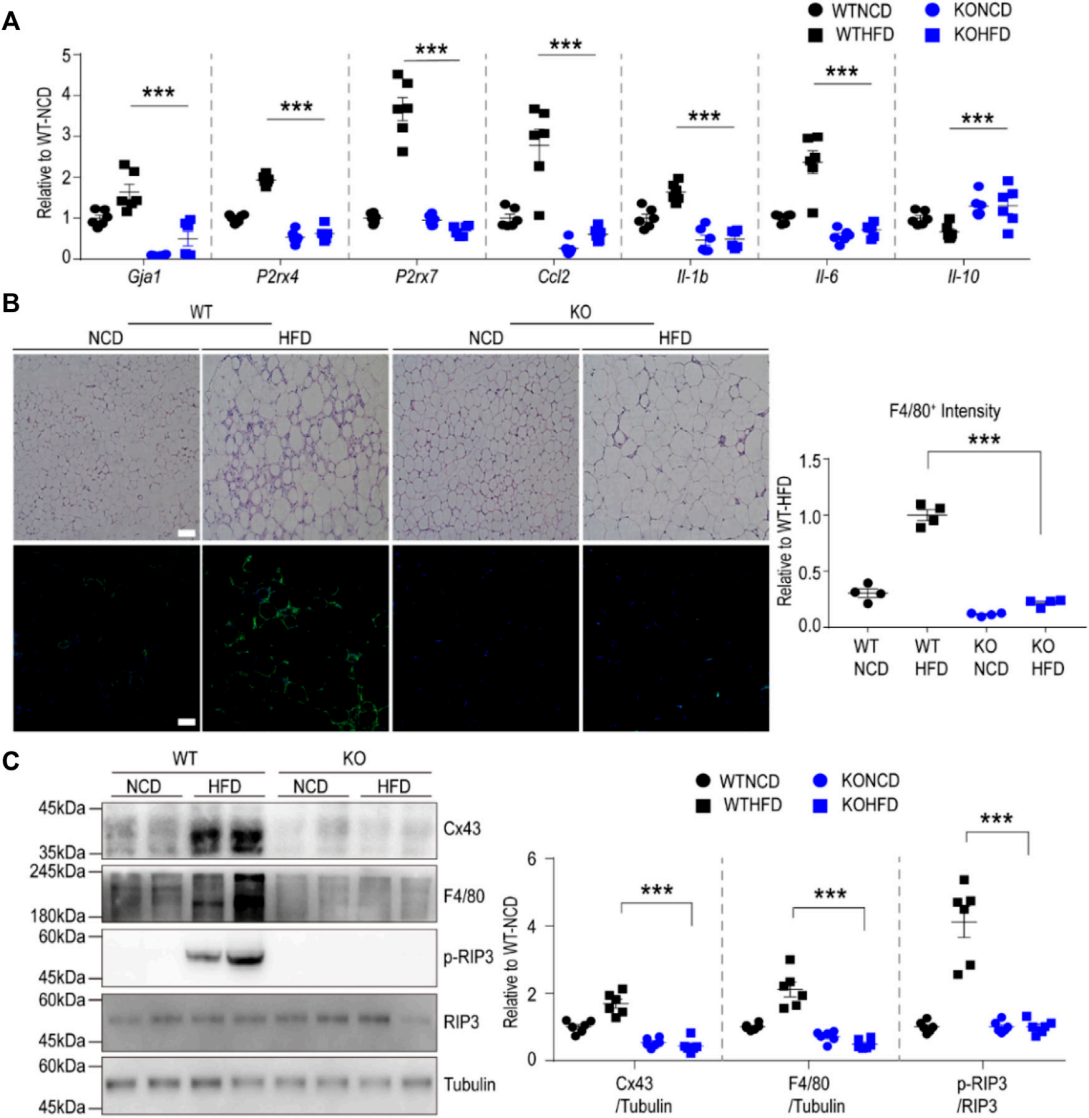
Macrophage-specific Cx43 KO protected mice from HFD-induced inflammation in gonadal white adipose tissue **(A)** qPCR analysis of gWAT of WT and Cx43 MKO mice fed with either normal chow diet (NCD) or high-fat diet (HFD) for 8weeks (n = 6). Significant effects of genotype (*Gja1*: *p =* 0.001, *P2rx4*: *p* < 0.0001, *P2rx7*: *p* < 0.0001, *Ccl2*: *p* < 0.0001, *Il-1b*: *p* < 0.0001, *Il-6*: *p* < 0.0001, *Il-10*: *p* < 0.0001) and significant effects of diet (*Gja1*: *p* < 0.0001, *P2rx4*: *p* < 0.0001, *P2rx7*: *p* < 0.0001, *Ccl2*: *p* < 0.0001, *Il-1b*: *p =* 0.0039, *Il-6*: *p* < 0.0001, *Il-10*: *p =* 0.0391) were observed. Significant differences between WT HFD and KO HFD groups were determined by Bonferroni post hoc tests **(B)** Representative images of H/E and F4/80 stained paraffin sections, and quantification of F4/80 fluorescence intensity in gWAT of WT and Cx43 MKO mice fed NCD or HFD for 8 weeks (n = 4). Significant effects of genotype (*p* < 0.0001) and diet (*p* < 0.0001) were observed. Significant differences between WT HFD and KO HFD groups were determined by Bonferroni post hoc tests **(C)** Immunoblot analysis of gWAT of WT and Cx43 MKO mice fed with either NCD or HFD for 8 weeks (*n* = 6). Significant effects of genotype (Cx43: *p* < 0.0001, F4/80: *p* < 0.0001, p-RIP3: *p* < 0.0001) and significant effects of diet (Cx43: *p* < 0.0037, F4/80: *p* = 0.003, p-RIP3: *p* < 0.0001) were observed. Significant differences between WT HFD and KO HFD groups were determined by Bonferroni post hoc tests. Two-way ANOVA with Bonferroni post hoc tests was used (***p < 0.001). Each point represents biological replicate. Data are presented as mean ± S.E.M.

**FIGURE 6:**
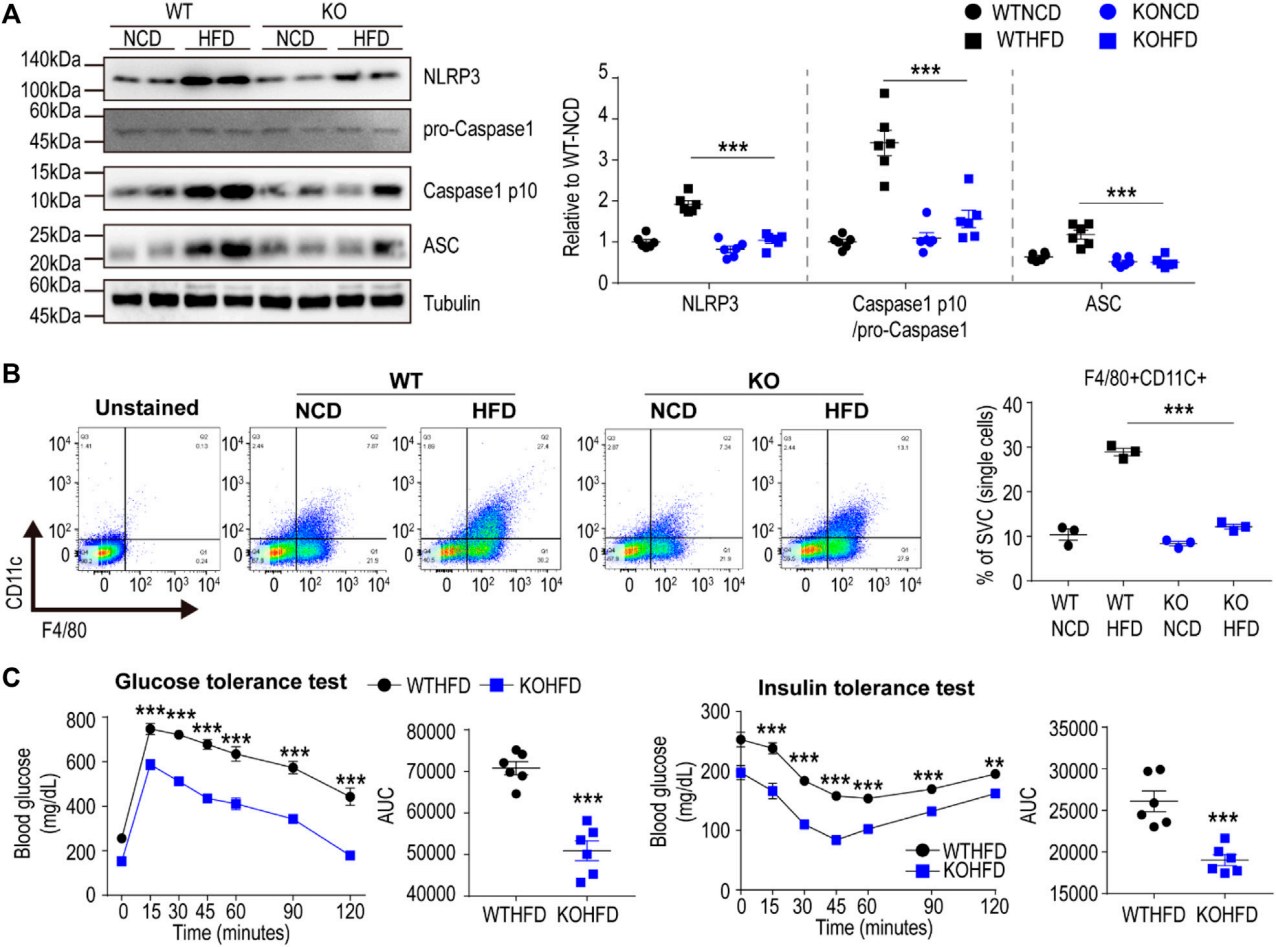
Macrophage-specific Cx43 KO protected mice from HFD induced metabolic dysfunction **(A)** Immunoblot analysis of gonadal white adipose tissue (gWAT) of WT and Cx43 MKO mice fed with normal chow diet (NCD) or high-fat diet (HFD) for 8 weeks (n = 6). Significant effects of genotype (NLRP3: *p* < 0.0001, Caspase1 p10: *p* < 0.0001, ASC: *p* < 0.0001) and significant effects of diet (NLRP3: *p* < 0.0001, Caspase1 p10: *p* = 0.0001, ASC: *p* = 0.0006) were observed. Significant differences between WT HFD and KO HFD groups were determined by Bonferroni post hoc tests **(B)** Flow cytometric analysis of SVCs obtained from gWAT of WT and Cx43 MKO mice fed with NCD or HFD for 8 weeks (n = 3). Significant effects of genotype (*p* < 0.0001) and diet (*p* < 0.0001) were observed. Significant differences between WT HFD and KO HFD groups were determined by Bonferroni post hoc tests **(C)** Glucose tolerance and insulin tolerance tests of WT and Cx43 MKO mice fed with HFD for 8 weeks (n = 6). Two-way ANOVA with Bonferroni post hoc tests was used in A-B. Unpaired, two-tailed t-tests (**p < 0.01, ***p < 0.001) were used in C. Each point represents biological replicate. Data are presented as mean ± S.E.M.

We further validated the RNA-seq data at transcript and protein levels using qPCR, immunoblotting, and immunohistochemical analyses. Based on qPCR analysis, HFD feeding upregulated P2RX7 and P2RX4 expression levels in the gWAT of WT mice, but not in the gWAT of Cx43-MKO mice (Figure 5A). Histological analysis of H&E-stained paraffin sections indicated that Cx43-MKO reduced the accumulation of crown-like structures in gWAT after HFD feeding (Figure 5B). Western blot analysis further confirmed that Cx43-MKO reduced HFD-induced recruitment of F4/80+ macrophages (Figure 5C). qPCR analysis confirmed the RNA-seq data, presenting reduced mRNA expression of pro-inflammatory cytokines in Cx43-MKO mice under HFD when compared with WT-HFD-fed mice (Figure 5A). It is well-established that P2RX7 signaling in macrophages facilitates the inflammasome signaling pathway. Consequently, inflammasome marker expression was decreased in the gWAT of MKO mice fed an HFD for 8 weeks (Figure 6A). Moreover, FACS analysis indicated that pro-inflammatory macrophages (F4/80+CD11c+) were reduced in KO mice fed an HFD (Figure 6B). Phenotypic analysis revealed that Cx43-MKO mice exhibit improved glucose tolerance and insulin sensitivity (Figure 6C).

## 4 Discussion

Obesity and related metabolic diseases can be characterized by a chronic inflammatory status (Reilly and Saltiel, 2017). Changes in macrophage phenotypes are correlated with the pathological remodeling of adipose tissue, thereby contributing to the development of insulin resistance and obesity-related metabolic dysfunction (Reilly and Saltiel, 2017).

Cx43 plays a key role in gap junction communication between cells (Evans and Martin, 2002). In addition to functioning as a gap junction channel, Cx43 hemichannels can mediate ATP release from cells and consequently regulate the autocrine activation of macrophages via ATP signaling (Dosch et al., 2019). Consistently, our data indicated that macrophage expression of Cx43 is critical during extracellular ATP signaling for pro-inflammatory macrophages activation in adipose tissue during HFD feeding.

Interestingly, we observed that macrophage-specific deletion of Cx43 protected mice from HFD-induced inflammation, consequently ameliorating glucose tolerance and insulin sensitivity. It is well-known that inflammatory responses in adipose tissue are one of the primary factors that contribute to the development of over-nutrition-induced metabolic dysfunction. In this regard, adipose tissue macrophages have been characterized as the major cell types responsible for pro-inflammatory cytokine production and inflammation. For instance, in patients with obesity, hypertrophic adipose tissue is frequently associated with pro-inflammatory macrophages and crown-like structures. Previous studies using diet-induced obesity mouse models reported that gWAT manifests more prominent macrophage infiltration, compared to other depots at different anatomical locations, such as subcutaneous and mesenteric WAT (van Beek et al., 2015). Thus, in this study, we focused on gWAT to investigate macrophage-specific roles of Cx43 during the development of obesity in mice.

We hypothesized that Cx43-mediated ATP release and the purinergic receptor P2RX7 signaling pathway in macrophages are factors that facilitate over-nutrition-induced inflammatory responses. This observation further suggests that pharmacological inhibition of Cx43 could afford a novel therapeutic strategy for obesity-associated inflammation and resistance. However, although the current study focused on the roles of macrophage-expressed Cx43, it is crucial to note that Cx43 activation in adipocytes mediates beneficial effects by facilitating cAMP coupling between adipocytes to increase protein kinase A (PKA)-signaling-mediated lipid catabolism and energy expenditure (Zhu et al., 2016). Therefore, the cell type-specific delivery of Cx43 modulators is required to develop novel therapies to treat obesity-related metabolic diseases.

Herein, our findings suggest that Cx43 KO inhibits the P2RX7-mediated activation of macrophages. Although not examined in the current study, extracellular ATP signaling is also known to be involved in regulating adipocyte function. For example, P2X7 receptor downstream signaling regulates lipid metabolism and adipogenesis, and P2RX7 KO affects fat distribution *in vivo* (Beaucage et al., 2014). In the present study, we could not precisely determine the cell type-specific contribution of Cx43/ATP/P2RX7 purinergic signaling. Further studies using single-cell level analysis are necessary to comprehensively clarify the effects of macrophage-specific Cx43 KO on other cell types, including adipocytes, through paracrine mechanisms.

Collectively, our study demonstrates the critical role of Cx43 in the pro-inflammatory activation of macrophages during HFD-induced adipose tissue remodeling in mice. Mechanistically, Cx43-mediated ATP release could induce autocrine macrophage activation, potentially through P2RX7. Identifying molecular players in over-nutrition-induced macrophage activation is critical for understanding adipose tissue inflammation and insulin resistance.

## Data Availability

The datasets presented in this study can be found in online repositories. The names of the repository/repositories and accession number(s) can be found below: NCBI GEO, accession no: GSE204794.
