# Supplementary material for: Macrophage-Specific Connexin 43 Knockout Protects Mice from Obesity-Induced Inflammation and Metabolic Dysfunction
Source: Front Cell Dev Biol. 2022 Jun 21;10:925971. doi: 10.3389/fcell.2022.925971 (PMC9253378; doi:10.3389/fcell.2022.925971)
Supplement: Supplementary file 1 [file DataSheet1.PDF]

Supplementary Material

Supplemental figures

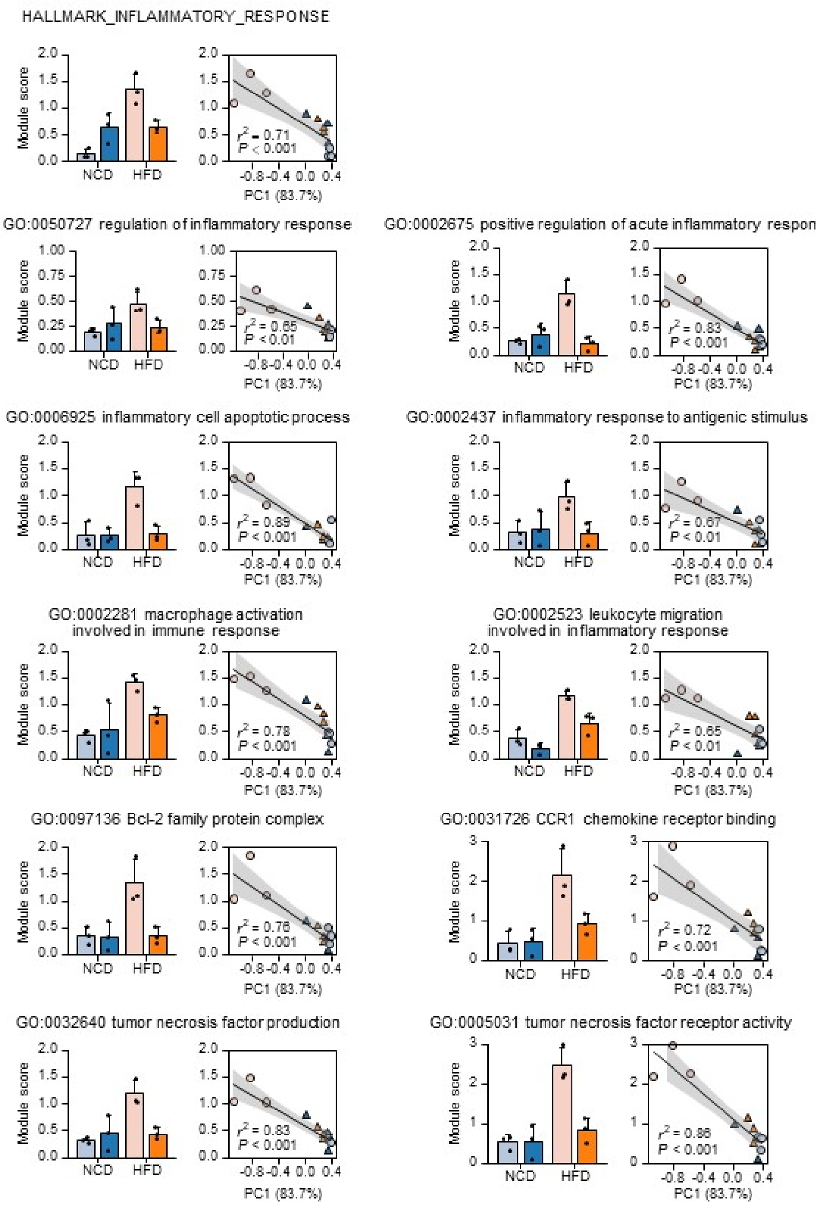

Figure S1. Module score and correlation analysis of genes involved in inflammation

Module scores of genes associated with inflammatory response were calculated by averaging Z-normalized expression. Correlation analysis between module score of genes associated with inflammatory response and PC1 values (from Figure 3B). Genes involved in inflammation were positively regulated in WT-HFD (average expression), whereas not in other groups. PC1 showed a high correlation with the average expression of genes associated with each enrichment analysis, confirming that it is a major biological process that differentiates WT-HFD group from other groups.

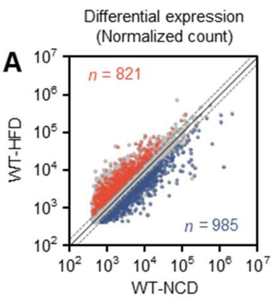

ID	Term name	Gene symbol	–log*P*
WT-HFD UP (vs WT-NCD)			
GO:0032675	regulation of interleukin-6 production	App,Csk,Cyba,Fcer1g,Lilr4b,Lilrb4a,Ptpn6,Il16,Il6ra,Inpp5d,Laptm5,Arhgef2,Lbp,Ptafr,Sirpa,Syk,Tlr4,Tnfaip3,Tyrobp,Irak3,Nckap1l,Tlr3,Tlr7,Tlr8,Tmem106a,Cd300ld,Afap1l2,Rab7b,Zc3h12a,Plcg2	11.39
GO:1902105	regulation of leukocyte differentiation	Zfp36l1,Zfp36l2,C1qc,Cbfb,Cdk6,Ctla2a,Fbn1,Fes,Fos,Lilr4b,Lilrb4a,Hcls1,Ptpn6,Hlx,Inpp5d,Mafb,Loxl3,Lyn,Mitf,Ninj1,Ptprc,Syk,Tgfb1,Tgfbr2,Klf10,Tyrobp,Axl	9.63
GO:0042117	monocyte activation	Adam10,Adam9,Fn1,Lilr4b,Lilrb4a	4.95
GO:0150146	cell junction disassembly	Arf6,C1qa,C1qb,C1qc	4.27
WT-HFD DOWN (vs WT-NCD)			
GO:0006084	acetyl-CoA metabolic process	Cs,Dld,Fasn,Mvk,Pdha1,Pdhx,Mpc1,Acss2,Nudt7,Pdhb,Pmvk,Mpc2,Acacb,Acly,Acaca,Acat1,Mvd,Pdk1,Dlat	14.23
GO:0019395	fatty acid oxidation	Acadvl,Acads,Akt2,Cpt2,Crat,Eci1,Dgat1,Hadh,Phyh,Por,Abcd3,Scp2,Slc25a17,Echdc2,Echdc1,Ivd,Etfdh,Lonp2,Decr1,Dgat2,Tysnd1,Pex13,Ehhadh,Echs1,Acacb,Acat1,Acat2	13.33
GO:0006119	oxidative phosphorylation	Atp5a1,Atp5b,Atp5c1,Coq7,Dld,Ndufv1,Uqcrc1,Mtch2,Mlxipl,Ndufa12,Cyc1,Stoml2,Sdhd,Sdha,Slc25a23,Ndufa10,Coq9,Ndufv3,Ppif,Proca1,Ndufs2,Ndufs1	7.88
GO:0006734	NADH metabolic process	Gpd1,Gpd2,Idh3g,Ldha,Mdh2,Mdh1,Pfkl,Pfkm	6.42

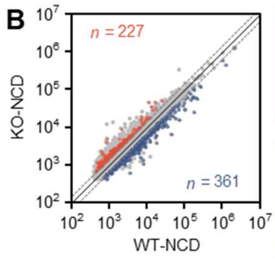

ID	Term name	Gene symbol	–log*P*
KO-NCD UP (vs WT-NCD)			
GO:1902105	regulation of leukocyte differentiation	Zfp36l2,Cd83,Cdk6,Csf1,Csf1r,Fbn1,Hlx,Id2	5.14
GO:0038145	macrophage colony-stimulating factor signaling pathway	Csf1,Csf1r	2.05
GO:0045446	endothelial cell differentiation	Col18a1,Dll1,Fstl1,S1pr2	1.85
GO:0002573	myeloid leukocyte differentiation	Cdk6,Csf1,Csf1r,Fbn1,Id2,Sfrp1,Tgfbr2	1.83
GO:0022409	positive regulation of cell-cell adhesion	Adam19,Cd83,Cd86	1.50
KO-NCD DOWN (vs WT-NCD)			
GO:0019395	fatty acid oxidation	Acadvl,Akt2,Cpt2,Dgat1,Hadh,Hsd17b4,Scp2,Slc25a17,Echdc1,Lonp2,Dgat2,Adipor2,Tysnd1,Ehhadh,Echs1,Acacb,Acat2	10.49
KEGG:03320	PPAR signaling pathway	Plin2,Cpt2,Acsl1,Lpl,Me1,Rxrg,Scd1,Scd2,Scp2,Nr1h3,Slc27a4	5.86
GO:0019432	triglyceride biosynthetic process	Dgat1,Acsl1,Lpl,Thrsp,Nr1h3,Dgat2	3.14
GO:0000041	transition metal ion transport	Fkbp4,Hfe,Slc11a2,Slc31a1,Trf,Tfrc	2.00
GO:0006006	glucose metabolic process	Akt2,Gapdh,Gpd1,Igf1,Ldha,Pfkl,Rbp4,Tpi1	2.32

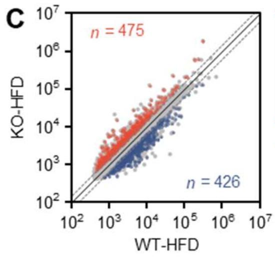

ID	Term name	Gene symbol	–log*P*
KO-HFD UP (vs WT-HFD)			
GO:0009083	branched-chain amino acid catabolic process	Bcat2,Ivd,Acadsb,Mccc2,Aldh6a1,Acat1,Hibch	5.52
GO:0050873	brown fat cell differentiation	Arl4a,Cebpa,Scd1,Selenbp1,Six1,Slc2a4,Sox13,Nudt7,Aldh6a1,Ffar4	4.57
GO:0016042	lipid catabolic process	Acads,Adora1,Akt2,Hadh,Lipe,Thra,Mgll,Echdc2,Pla2g6,Ivd,Abhd6,Pla2g12a,Nudt7,Echs1,Plin1,Ces1d,Acat1,Acat2	3.48
GO:0008203	cholesterol metabolic process	Cebpa,Fdft1,Lss,Mvk,Nsdhl,Srebf1,Pmvk,Dhcr24,Ces1d,Ces1f,Sc5d	1.93
GO:0120161	regulation of cold-induced thermogenesis	Cav1,Cmklr1,Hadh,Npr3,Scd1,Thra,Tshr,Abhd6,Ffar4,Elovl6,Nr1d1	1.55
KO-HFD DOWN (vs WT-HFD)			
GO:0002274	myeloid leukocyte activation	Adam10,Cd48,Fcer1g,Fes,Lilrb4a,Grn,Ptpn6,Hmox1,Ifngr2,Slc11a1,Pirb,Rbpj,Spi1,Syk,Tlr4,Tyrobp	7.48
GO:0050764	regulation of phagocytosis	Csk,Cyba,Fcer1g,Itgav,Cd47,Slc11a1,Pros1,Sirpa,Syk,Pot1b,Nckap1l,Abr,Plcg2	4.66
GO:0050729	positive regulation of inflammatory response	Alox5ap,Ctss,Fcer1g,Grn,Cd47,Ninj1,Ptger4,Tlr4,Tnfrsf11a,Pik3cg	3.99
GO:0030595	leukocyte chemotaxis	Adam10,Adam17,C3ar1,Fcer1g,Ninj1,Lgmn,Dusp1,Ccl9,Spi1,Syk,Pla2g7,Vav3	2.85
GO:0061082	myeloid leukocyte cytokine production	Bcl6,Fcer1g,Hmox1,Laptm5,Tlr4	2.46

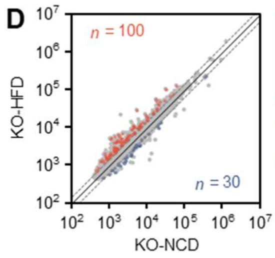

ID	Term name	Gene symbol	–log*P*
KO-HFD UP (vs KO-NCD)			
GO:0055117	regulation of cardiac muscle contraction	Smad7,Pde4b,Pkp2,Ankrd39,Pde4d	1.87
GO:0022409	positive regulation of cell-cell adhesion	Cd59a,Gcnt2,Lep,Smad7,Pck1,Dock8,Fstl3,Pde4d	1.66
GO:1901898	negative regulation of relaxation of cardiac muscle	Pde4b,Pde4d	1.53
GO:0006114	glycerol biosynthetic process	Lep,Pck1	1.42
KO-HFD DOWN (vs KO-NCD)			
KEGG:04015	Rap1 signaling pathway	Angpt1,Cnr1,Igf1	1.76
WP:WP447	Adipogenesis genes	Cfd,Igf1,Irs1	1.45

Figure S2. Differential expression analysis and enrichment analysis

(A) WT-HFD vs WT-NCD. (B) KO-NCD vs WT-NCD. (C) KO-HFD vs WT-HFD. (D) KO-HFD vs KO-NCD. (left) The normalized counts of each group were plotted, and DEGs satisfying fold change > 1.5 and P < 0.05 were presented in red and blue in case of up-regulated and down-regulated, respectively. The number of DEGs were labeled. (right) Significantly enriched gene set terms were presented with gene members of each set.

Supplementary Tables

Table S1. Antibodies used for Western blot and Immunohistochemistry

Antibody	Host	Company	Catalog #	Dilution
Cx43	Rabbit	Cell Signaling	3512	1:1000(WB)<br>1:100(IHC)
F4/80	Rabbit<br>Rat	Cell Signaling<br>Serotech	30325<br>MCA497GA	1:1000(WB)<br>1:100(IHC)
p-RIP3	Rabbit	Abcam	ab195117	1:1000
RIP3	Mouse	Cell Signaling	15828	1:1000
NLRP3/NLAP3	Mouse	AdipoGen Life Sciences	AG-20B-0014	1:1000
ASC/TMS1/PYCARD	Mouse	Santa Cruz	sc-514414	1:1000
Anti-Caspase1	Mouse	Santa Cruz	sc-56036	1:1000
α/β tubulin	Rabbit	Cell Signaling	2148	1:3000
Goar anti-rabbit IgG, HRP	Goat	Invitrogen	31460	1:3000
Goat anti-mouse IgG,HRP	Goat	Jackson	115-035-174	1:3000
Donkey anti-Rat IgG (H+L) Highly cross-adsorbed Secondary Antibody, Alexa Fluor^{TM} 488	Rat	Invitrogen	A21208	1:100 (IHC)

Table S2. Primers used for qPCR

Reverse primer	Forward primer	Primer
GTT TTC TCC GTG GGA CGT GA	CAG GTC TGA GAG CCC GAA CT	*Gja1*
CCG ACA GCA CGA GGC TTT	CTG GTG TGT GAC GTT CCC ATT A	*Il-1b*
TCT GAC CAC AGT GAG GAA TG	AGT GGC TAA GGA CCA AGA CC	*Il-6*
AAG GCA TCA CAG TCC GAG TC	CTG GAT CGG AAC CAA ATG AG	*Ccl2*
TCA CCT GCT CCA CTG CCT TGC	GGC AGA GAA GCA TGG CCC AGA A	*Il-10*
GTC AAA CTT GCC AGC CTT TCC	ACA ACG TGT CTC CTG GCT ACA AT	*P2rx4*
CCC CAC CCT CTG TGA CAT TCT	AGC ACG AAT TAT GGC ACC GT	*P2rx7*
TTA CAG GAC ATT GCG AGC AG	GTG GTC TTT GGG AAG GTG AA	*Ppia*

Table S3. Related to Figure 4. GSEA showing the enriched gene set that differentiates WT-HFD and the others

**Gene set**	**Size**	**ES**	**NES**	**NOM p-val**	**FDR q-val**	**q < 0.05**
HALLMARK_ALLOGRAFT_REJECTION	118	0.7861	1.8556	0.0000	0.0009	TRUE
HALLMARK_EPITHELIAL_MESENCHYMAL_TRANSITION	141	0.7452	1.7972	0.0000	0.0074	TRUE
HALLMARK_COMPLEMENT	127	0.7362	1.7540	0.0024	0.0131	TRUE
HALLMARK_INTERFERON_GAMMA_RESPONSE	167	0.7171	1.7532	0.0000	0.0098	TRUE
**HALLMARK_INFLAMMATORY_RESPONSE**	**106**	**0.7177**	**1.7043**	**0.0021**	**0.0208**	**TRUE**
HALLMARK_IL6_JAK_STAT3_SIGNALING	59	0.7720	1.6895	0.0194	0.0205	TRUE
HALLMARK_INTERFERON_ALPHA_RESPONSE	86	0.7402	1.6734	0.0042	0.0213	TRUE
HALLMARK_APICAL_JUNCTION	129	0.6591	1.6042	0.0109	0.0410	TRUE
HALLMARK_P53_PATHWAY	157	0.5379	1.3238	0.0792	0.2889	
HALLMARK_TNFA_SIGNALING_VIA_NFKB	138	0.5502	1.3158	0.1060	0.2724	
HALLMARK_KRAS_SIGNALING_UP	126	0.5478	1.3135	0.0869	0.2510	
HALLMARK_IL2_STAT5_SIGNALING	137	0.5298	1.2603	0.1484	0.3020	
HALLMARK_TGF_BETA_SIGNALING	50	0.5486	1.1580	0.2681	0.4455	
HALLMARK_KRAS_SIGNALING_DN	38	0.5192	1.0915	0.3457	0.5465	
HALLMARK_HEDGEHOG_SIGNALING	18	0.5734	1.0802	0.3834	0.5305	
HALLMARK_PROTEIN_SECRETION	89	0.4275	0.9792	0.5145	0.7137	
HALLMARK_UNFOLDED_PROTEIN_RESPONSE	107	0.4022	0.9328	0.5689	0.7833	
HALLMARK_HEME_METABOLISM	141	0.3789	0.9058	0.6114	0.8057	

HALLMARK_APICAL_SURFACE	26	0.4346	0.8619	0.6442	0.8631
HALLMARK_PANCREAS_BETA_CELLS	12	0.4751	0.8574	0.6319	0.8287
HALLMARK_G2M_CHECKPOINT	137	0.3305	0.8018	0.8322	0.9017
HALLMARK_E2F_TARGETS	141	0.2904	0.7105	0.9604	0.9984
HALLMARK_WNT_BETA_CATENIN_SIGNALING	29	0.3045	0.6169	0.9229	1.0000
HALLMARK_NOTCH_SIGNALING	29	0.2601	0.5283	0.9878	0.9947

GSEA was performed against hallmark gene sets (h.all.v7.5.symbols). Size: the number of genes in the corresponding gene set; ES: enrichment score; NES: normalized ES; NOM p-val: nominal *P* value; FDR q-val: false discovery rate-adjusted *q* value; $q < 0.05$: significance threshold.

Full-length blots for figures

Figure1A

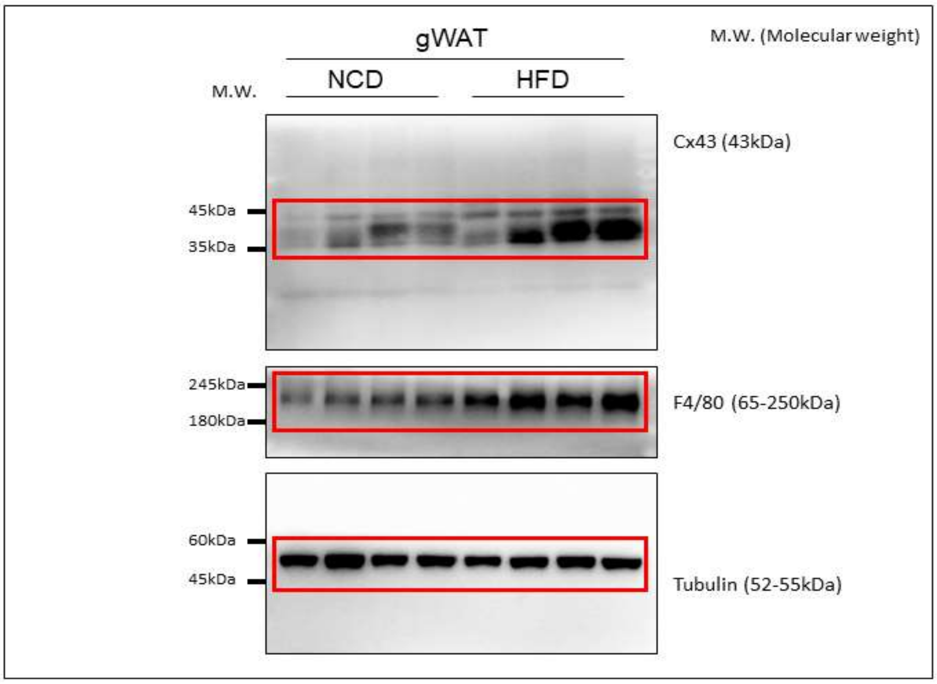

Figure2A

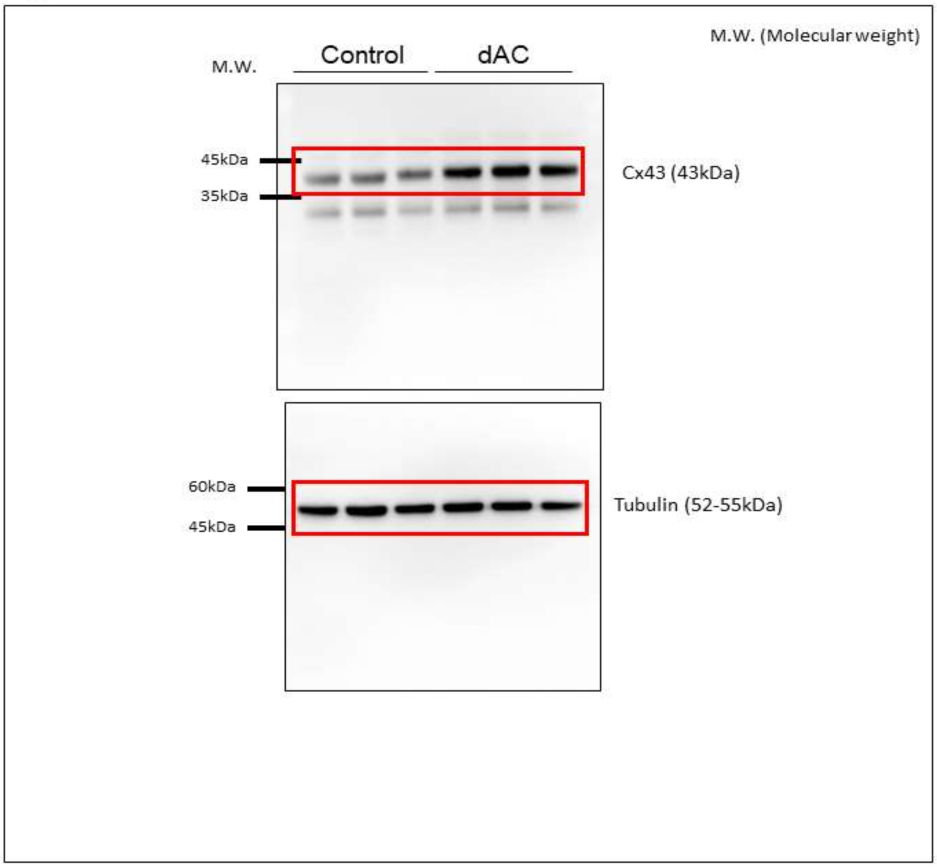

Figure5C

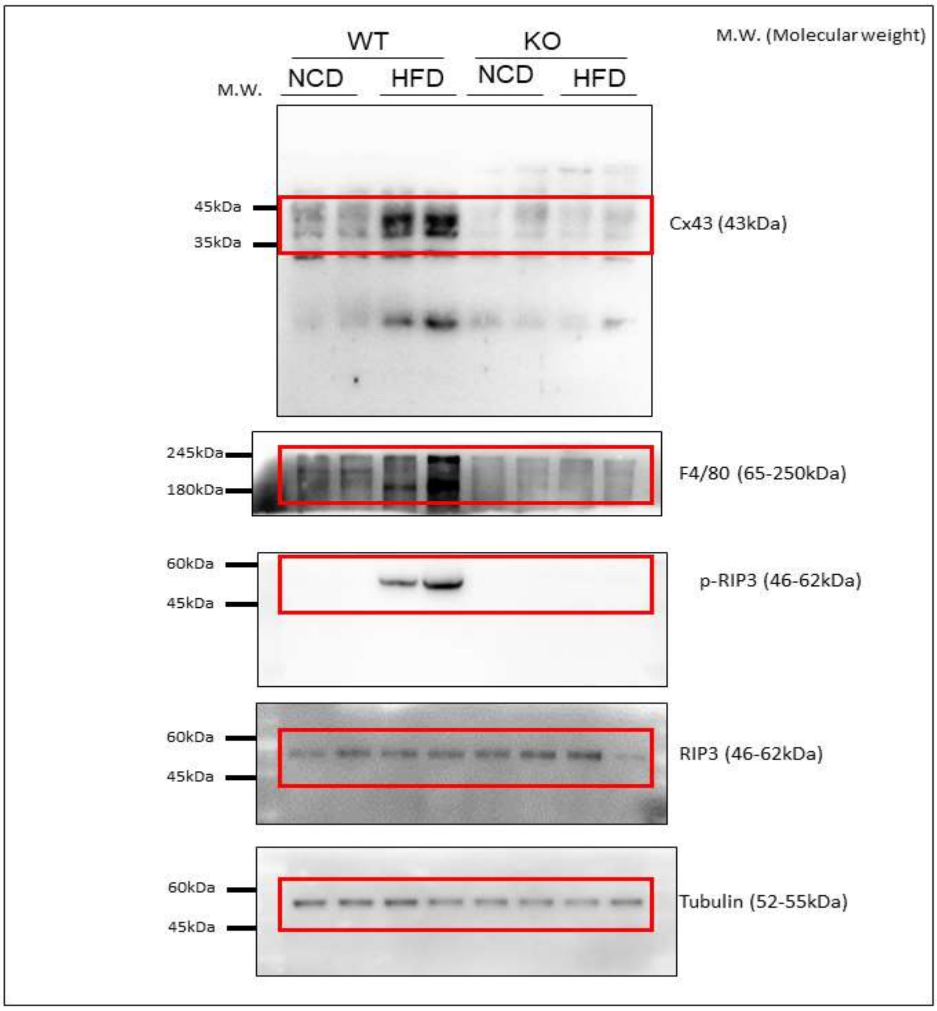
WT
KO
M.W. (Molecular weight)
M.W.
NCD
HFD
NCD
HFD
45kDa
35kDa
Cx43 (43kDa)
245kDa
180kDa
F4/80 (65-250kDa)
60kDa
45kDa
p-RIP3 (46-62kDa)
60kDa
45kDa
RIP3 (46-62kDa)
60kDa
45kDa
Tubulin (52-55kDa)

Figure6A

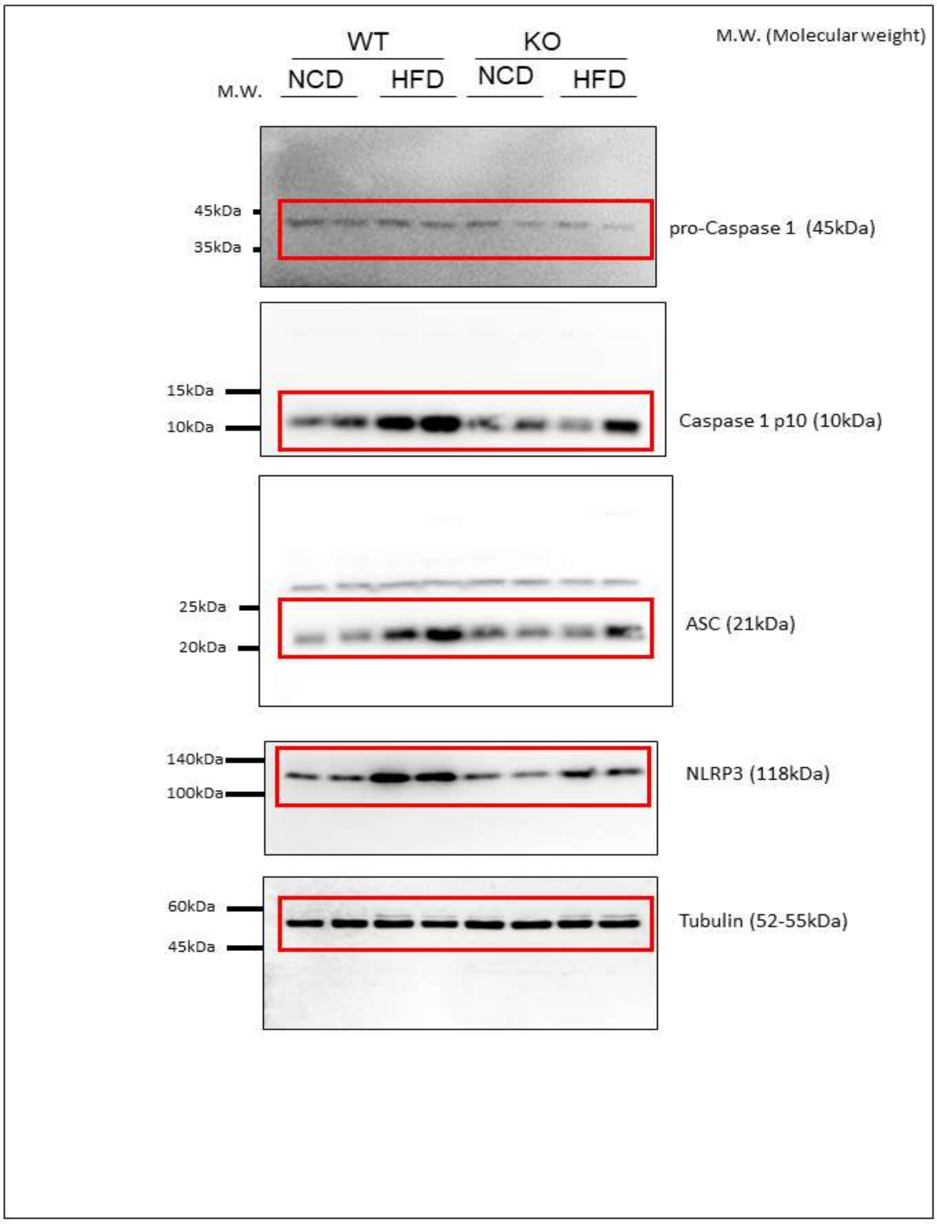
WT
KO
M.W. (Molecular weight)
M.W.
NCD
HFD
NCD
HFD
45kDa
35kDa
pro-Caspase 1 (45kDa)
15kDa
10kDa
Caspase 1 p10 (10kDa)
25kDa
20kDa
ASC (21kDa)
140kDa
100kDa
NLRP3 (118kDa)
60kDa
45kDa
Tubulin (52-55kDa)
